# Chain stopper engineering for hydrogen bonded supramolecular polymers

**DOI:** 10.3762/bjoc.6.102

**Published:** 2010-09-21

**Authors:** Thomas Pinault, Bruno Andrioletti, Laurent Bouteiller

**Affiliations:** 1 UPMC Université Paris 06, UMR 7610, Chimie des Polymères, F-75005 Paris, France; 2 CNRS, UMR 7610, Chimie des Polymères, F-75005 Paris, France; 3 Université Claude Bernard-Lyon 1, ICBMS-UMR 5246, 43 Boulevard du 11 Novembre 1918, F-69622 Villeurbanne Cedex, France

**Keywords:** chain stopper, gel, hydrogen bond, supramolecular polymer, urea

## Abstract

Supramolecular polymers are linear chains of low molar mass monomers held together by reversible and directional non-covalent interactions, which can form gels or highly viscous solutions if the self-assembled chains are sufficiently long and rigid. The viscosity of these solutions can be controlled by adding monofunctional compounds, which interact with the chain extremities: chain stoppers. We have synthesized new substituted ureas and thioureas and tested them as chain stoppers for a bis-urea based supramolecular polymer. In particular, the bis-thiourea analogue of the bis-urea monomer is shown not to form a supramolecular polymer, but a good chain stopper, because it is a strong hydrogen bond donor and a weak acceptor. Moreover, all substituted ureas tested reduce the viscosity of the supramolecular polymer solutions, but the best chain stopper is obtained when two hydrogen bond acceptors are placed in the same relative position as for the monomer and when no hydrogen bond donor is present.

## Introduction

Supramolecular polymers are linear chains of low molar mass monomers held together by reversible and highly directional non-covalent interactions [1–3]. Because of their macromolecular architecture, they can display polymer-like rheological properties, and they can, in particular, form gels if the self-assembled chains are sufficiently long and rigid [4–9]. Compared to the well-known organogelators formed by the entanglement of usually crystalline fibers [10–13], supramolecular polymers display some specific features. In particular, hydrogen-bonded supramolecular polymers are often dynamic at room temperature, which means that they do not need to be heated and then cooled to form a gel. Moreover, the gels formed are usually visco-elastic, meaning that they show an elastic response only at high frequencies.

**Figure 1:**
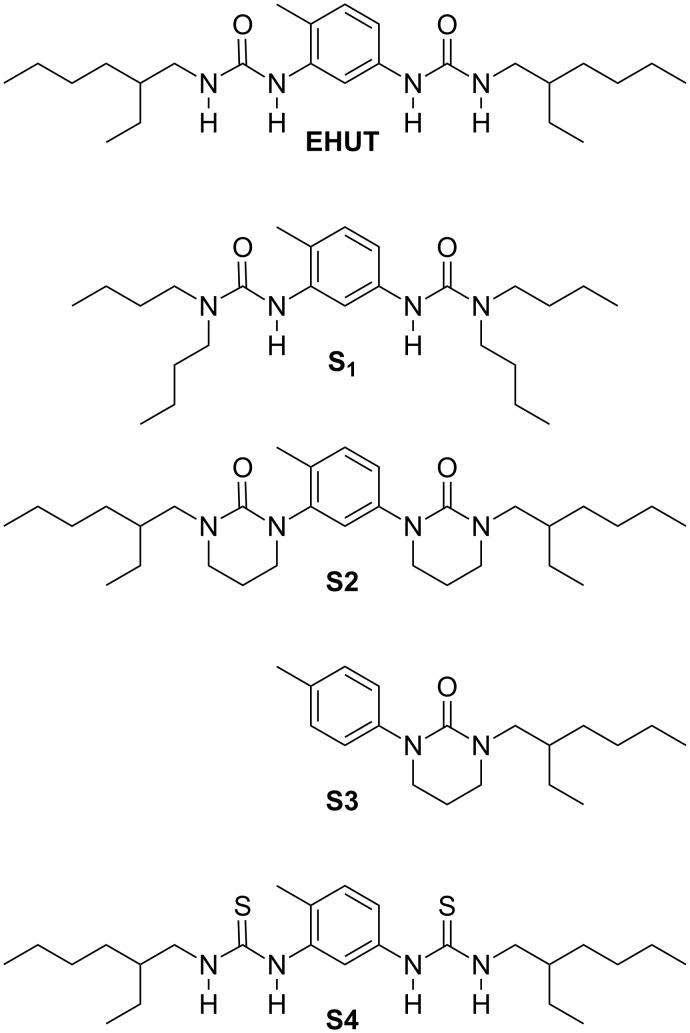
Structures of monomer **EHUT** and chain stoppers.

The chain length of a supramolecular polymer depends on the strength of the association between the monomers, which is highly dependent on their concentration, the temperature, the solvent, i.e., environmental factors, but also on the presence of additives. Chain-stoppers are monofunctional monomers able to interact with monomers and therefore able to break polymer chains. They can be introduced in order to reduce the length of the supramolecular polymer (and thus reduce the viscosity of the solution) [14–16], but also in order to block the concentration dependence of the supramolecular polymers [17–19]. Chain stoppers can also be exploited to decorate the chain-ends with particular functional groups or labels [20–21]. The effectiveness of these schemes depends directly on the design of the chain-stopper: the interaction between chain-stopper and monomer should ideally be as strong as the interaction between monomers. It is therefore of interest to identify chain stoppers with an improved affinity toward a given supramolecular polymer. In this article, we investigate the efficiency of several new chain stoppers for a well-known bis-urea-based supramolecular polymer **EHUT** (Figure 1). This supramolecular polymer is particularly interesting, because it has been previously shown to self-assemble cooperatively into two competitive high molecular weight structures [22–24].

## Results and Discussion

### Design and synthesis

The bis-urea based monomer **EHUT** has been shown to self-assemble in non-polar solvents, into two supramolecular polymeric structures, the tube or the filament forms, which are in dynamic exchange [23–24]. The respective stability of each form depends on the solvent, the temperature and the concentration. The filament form contains a single molecule in its cross section [25–27], and is the most stable structure at concentrations above 10^−3^mol/L and at room temperature, in solvents such as chloroform [22], carbon tetrachloride [28] and 1,3,5-trimethylbenzene [29]. The tube form contains three molecules in its cross section [6,30–31], and is the most stable structure at concentrations above 10^−5^mol/L and at room temperature, in solvents such as toluene [32] and dodecane [5].

**Figure 2:**
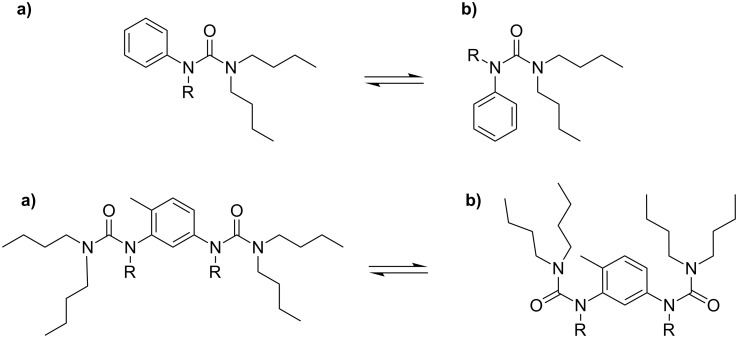
Substituted urea conformation. If R is alkyl, the most stable conformation is b); if R = H, the most stable conformation is a) [37].

Chain stopper **S1**, with two NH groups replaced by *N*-butyl groups, was previously shown to be a good chain stopper for **EHUT** in carbon tetrachloride [17], i.e., a good chain stopper of the filament form. However, at high concentrations, the two remaining NH groups were shown to form hydrogen bonds [17], and therefore **S1** can also behave to some extent as a co-monomer of **EHUT**: a small proportion of **S1** molecules may be incorporated in the filament structure rather than at its extremities. Simple alkylation of the 2 remaining NH groups does not yield an efficient chain stopper [17]. This surprising result was tentatively attributed to the conformation of the tetrasubstituted urea group, which may be ill-adapted to form hydrogen bonds to the urea groups of **EHUT** (Figure 2).

Hence, we introduced cyclic urea groups in the structure of chain stopper **S2**, by the alkylation of **EHUT** with 1,3-dibromopropane [33]. The rigidity of the cyclic ureas forbids any conformational rearrangement and should make it possible to probe whether the presence of NH functions in **S1** significantly affects the chain stopper efficiency. In order to see if both urea carbonyls in **S2** interact cooperatively with **EHUT** assemblies ,the mono-urea stopper **S3** was also prepared. Finally, chain stopper **S2** can only interact with bis-urea assemblies as a hydrogen bond acceptor through its carbonyl groups. It is therefore of interest to try and design a potentially complementary chain stopper, which would interact with bis-urea assemblies as a hydrogen bond donor. For this purpose, we synthesized the bis-thiourea **S4**, from the corresponding bis-thioisocyanate, because thioureas are known to be strong hydrogen bond donors and weak hydrogen bond acceptors [34–35].

Before evaluating the chain stopper efficiency of these compounds, i.e., their interaction with **EHUT**, their self-association was probed.

### Self-association of bis-thiourea

**Figure 3:**
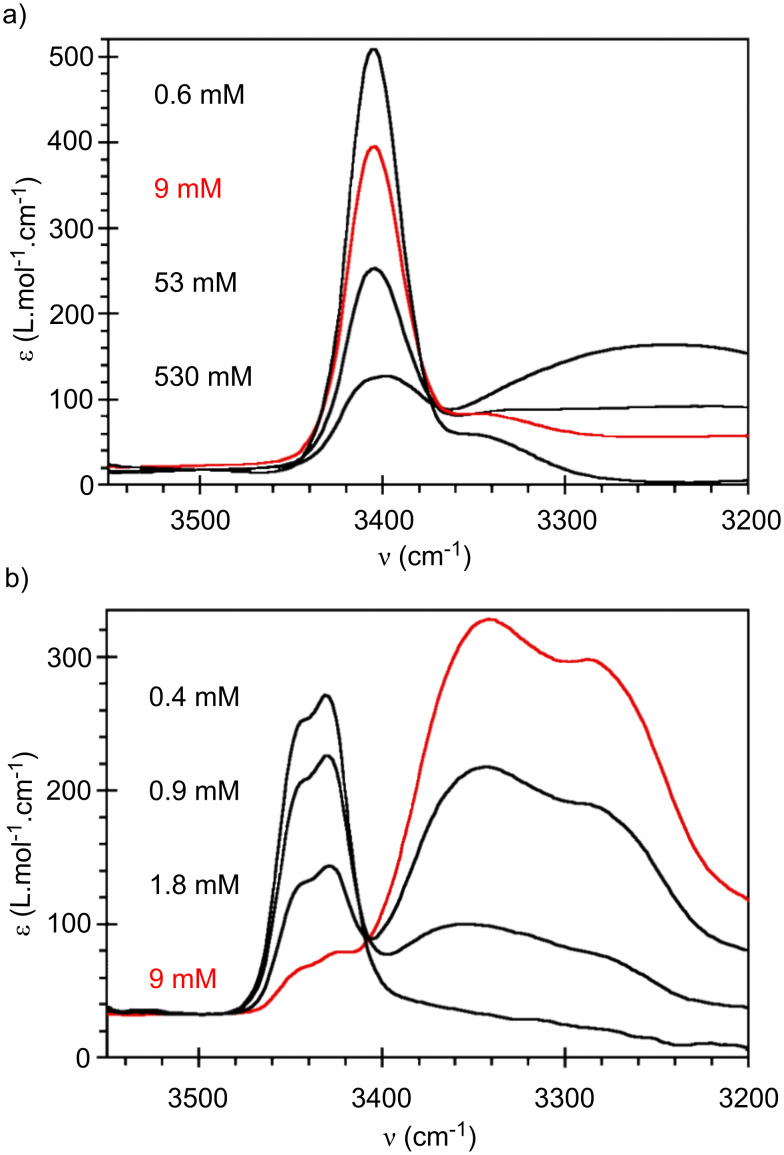
FTIR spectra, at 20 °C, for CDCl_3_ solutions of **S4** (a) or **EHUT** (b)**,** at several concentrations.

**Figure 4:**
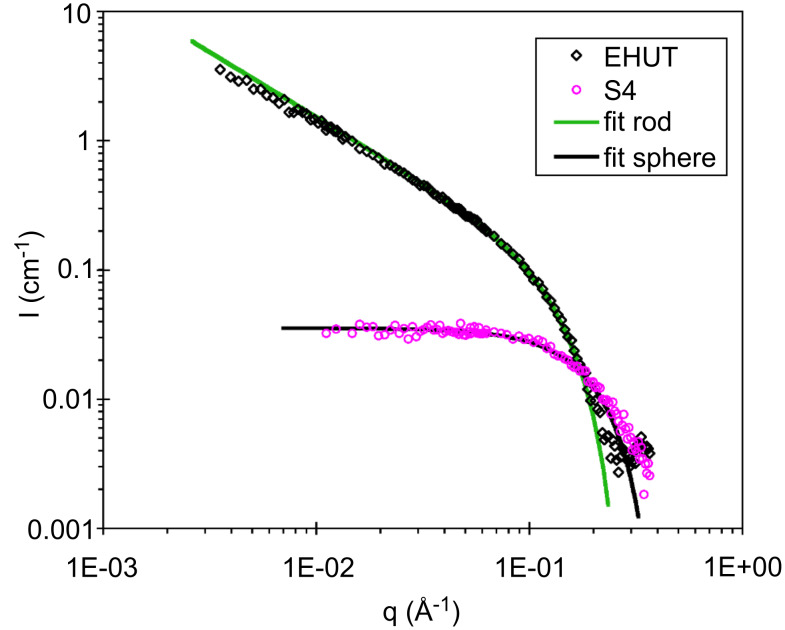
SANS intensity versus scattering vector for 12 mM solutions of **EHUT** or **S4** in *d*_8_-toluene, at 22 °C. The plain curves are fits according to a model for infinitely long rigid rods of diameter 26 Å (green), or for spheres of diameter 22 Å (black).

Chain stoppers **S2** and **S3** cannot self-associate because they contain only hydrogen bond acceptors, however this is not the case for **S4**, and it is of interest to determine the conditions under which **S4** can be considered not to associate with itself. Figure 3a shows the FTIR spectra of **S4** at several concentrations in chloroform. At concentrations below 53 mM, a single band is visible in the region corresponding to the N–H stretching vibration. This band (3405 cm^−1^) can be attributed to free NH groups. Only at a high concentration (0.5 mol/L) does a band characteristic for hydrogen bonded NH groups appear (3250 cm^−1^). The very weak hydrogen bonding propensity of bis-thiourea **S4** is particularly obvious when compared to bis-urea **EHUT** (Figure 3b): at the same concentration (9 mM), the bis-urea is nearly fully associated, whereas the bis-thiourea is virtually not associated. The respective behaviour of the bis-urea and the bis-thiourea was also probed by Small Angle Neutron Scattering (SANS) in toluene. Figure 4 shows the previously established q^−1^ dependence of **EHUT**, which is characteristic for long and rigid fibrillar scatterers [23]. In contrast, the low intensity and flat profil for **S4** at small angles is characteristic for small globular scatterers. A fit was performed with the form factor of a sphere, and yields a diameter of 22 Å, which is comparable to the largest dimension of the fully extended molecule (25 Å). In conclusion, bis-thiourea **S4** does not self-assemble significantly at concentrations below 12 mM in toluene or 53 mM in chloroform.

### Chain stopper effect on the EHUT filament structure

**Figure 5:**
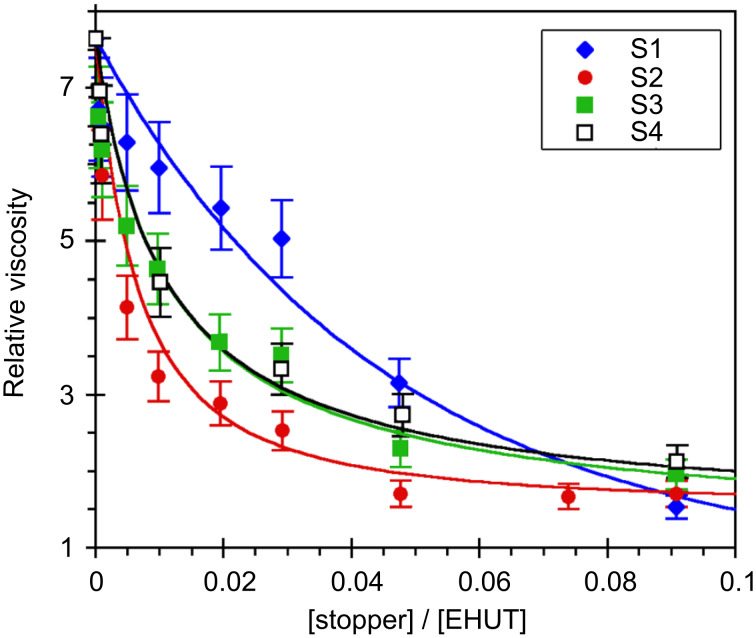
Relative viscosity for solutions of **EHUT** (20 mM) in 1,3,5-trimethylbenzene at 25 °C, with increasing molar fraction of chain stoppers. The lines are a guide for the eye only.

Viscosimetry is certainly the most sensitive technique to probe the efficiency of a chain stopper on supramolecular polymers. Therefore, we measured the viscosity of solutions of **EHUT** at a fixed concentration (20 mM) with increasing amounts of chain stopper. For this, 1,3,5-trimethylbenzene was chosen as the solvent because it is known to favor the formation of **EHUT** filaments at room temperature [29]. Figure 5 shows that all four compounds strongly reduce the relative viscosity of **EHUT**, which decreases from a value of 7.6 in the absence of stopper to a value close to 1 (i.e. the solution has approximately the same viscosity as the solvent) for a molar fraction ratio of stopper to monomer of 0.1. However, there are some significant differences between the stoppers: their efficiency increases in the order **S1** < **S3** ≈ **S4** < **S2**. Several conclusions can be derived from this result. First, the lower viscosity of solutions containing **S2** than those containing **S1** means that the remaining two NH groups of **S1** do participate in hydrogen bonding and reduce the efficiency of the stopper. Secondly, the lower viscosity of solutions containing **S2** than those containing **S3** indicates that both carbonyls are probably involved in the association between **S2** and an **EHUT** filament. Finally, bis-thiourea **S4** is a reasonably good chain stopper. The fact that it is not as good as **S2** is perhaps due to some marginal hydrogen bonding involving the thiocarbonyl groups.

### Chain stopper effect on the EHUT tube structure

**Figure 6:**
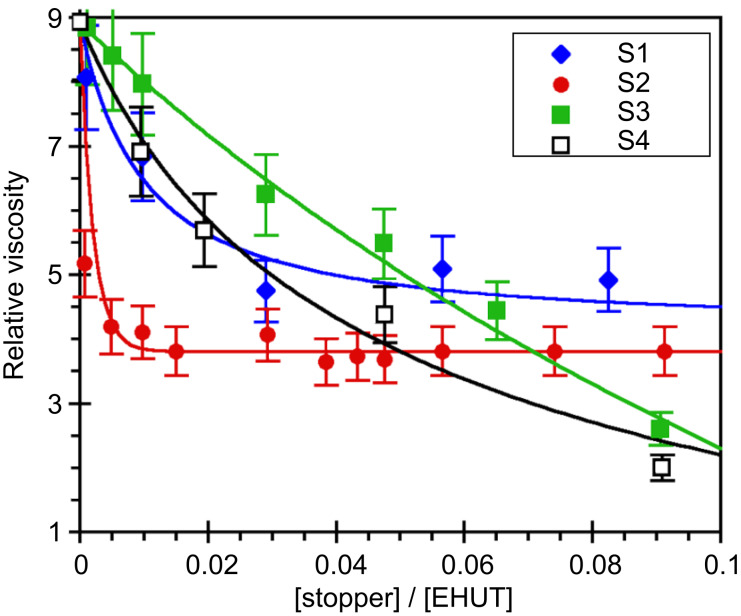
Relative viscosity for solutions of **EHUT** (1.1 mM) in toluene at 25 °C, with increasing molar fraction of chain stoppers. The lines are a guide for the eye only.

For the above, toluene was chosen as the solvent, because it is known to favor the formation of **EHUT** tubes at room temperature [32] and has a similar polarity as 1,3,5-trimethylbenzene. Figure 6 shows that all four compounds also reduce the relative viscosity of **EHUT** in toluene, but the situation is more complex than in trimethylbenzene. If we consider first the part of the curves with a stopper to monomer fraction lower than 0.05, the efficiency of the chain stoppers increases in the order **S3** < **S1** ≈ **S4** < **S2**. Therefore, the same conclusions for the interactions with the **EHUT** tubes can be derived as for the interactions with the **EHUT** filaments: i) the lower viscosity of solutions containing **S2** than those containing **S1** means that the remaining two NH groups of **S1** participate in hydrogen bonding and reduce the efficiency of the stopper; ii) the lower viscosity of solutions containing **S2** than those containing **S3** indicates that both carbonyl groups are involved in the association between **S2** and an **EHUT** tube; and iii) bis-thiourea **S4** is a reasonably good chain stopper, but not as good as **S2** probably due to some marginal hydrogen bonding involving the thiocarbonyls. If we consider now the part of the curves with a stopper to monomer fraction larger than 0.05, it is surprising to see that instead of the value decreasing to 1, the relative viscosity reaches a plateau at a value of 5 and 4 in the cases of **S1** and **S2**, respectively. To our knowledge, such a saturating effect is unprecedented, and may indicate that an additional mechanism is involved in the interaction between the bis-urea tubes and **S1** or **S2**. For example, we can hypothesize that at sufficiently high concentrations, **S1** or **S2** do not only interact with the extremities of the tubes, but also anywhere along them, without breaking them. However, additional characterizations will be required to test this interpretation [36].

## Conclusion

We have synthesized new substituted ureas and thioureas and tested them as chain stoppers for a bis-urea based supramolecular polymer. Depending on the solvent used, the bis-urea either forms filaments with a single monomer in the cross-section or tubes with three monomers in the cross-section. For both supramolecular architectures, similar conclusions can be derived: while all compounds tested reduce the viscosity of the supramolecular polymer solutions, the best chain stopper is obtained when two hydrogen bond acceptors are placed in the same relative position as for the monomer, and when no hydrogen bond donor is present.

Moreover, we have shown that a bis-thiourea with the same structure as the bis-urea monomer does not to form a supramolecular polymer, but acts as a good chain stopper, because it is a strong hydrogen bond donor and a weak acceptor.

## Experimental

### Synthesis

The synthesis of **EHUT** [32] and chain stopper **S1** [17] have previously been reported.

**Chain stopper S2**. NaH (9 g) was placed in a three necked round bottomed flask and washed with pentane (25 mL) under a nitrogen atmosphere. An **EHUT** solution (4.32 g, 10 mmol) in dry THF (400 mL) was added and the mixture stirred for 1 h. 1,3-Dibromopropane (20.5 mL, 200 mmol) in dry THF (100 mL) was then added, and the solution heated under reflux for 24 h. After cooling, ice was slowly added and the solvent evaporated. Chloroform (200 mL) was added and the organic phase washed successively with brine (300 mL) and water (2 × 300 mL), dried over magnesium sulfate and concentrated. Silica gel column chromatography (ethyl acetate) followed by recrystallization from pentane afforded 1.9 g of a white solid (37%). ^1^H NMR (200 MHz, DMSO-*d*_6_): δ (ppm) = 7.2 (d, *J* = 1.5 Hz, 1H, Ar-*H*), 7.12 (d, *J* = 8.1 Hz, 1H, Ar-*H*), 7.06 (dd, *J* = 8.1 Hz, *J* = 1.5, 1H, Ar-*H*), 3.74–3.02 (m, 12H, N-C*H*_2_), 2.21 (s, 3H, Ar-C*H*_3_), 2.07 (m, 4H, C*H*_2_), 1.66 (m, 2H, C*H*), 1.36 (m, 16H, C*H*_2_), 0.92 (t, 12H, C*H*_3_). ^13^C NMR (50 MHz, DMSO-*d*_6_): δ (ppm) = 153.7/153.2 (*C*=O), 136.3/136.2/129.9/129.7/108.3/103.2 (*Ar*), 51.7/51.5/50.3/50.2/47.7/46.9 (N-*C*H_2_), 37.4 (*C*H), 31.5/31.3/27.9/27.7/24.2/24.1/22.3 (*C*H_2_), 17.2 (Ar-*C*H_3_), 14/13.8/11.5/11.4 (*C*H_3_).

**Chain stopper S3.** NaH (1.5 g) was placed in a three necked round bottomed flask and washed with pentane (5 mL) under a nitrogen atmosphere. A solution of *N-*(2-ethylhexyl)-*N'*-(4-methylphenyl)urea [22] (1 g, 3.8 mmol) in dry THF (25 mL) was added and the mixture stirred for 1 h. 1,3-Dibromopropane (3.9 mL, 38 mmol) in dry THF (50 mL) was then added, and the solution heated under reflux for 24 h. After cooling, ice was slowly added and the solvent evaporated. Chloroform (50 mL) was added and the organic phase washed successively with brine (70 mL) and water (2 × 70 mL), dried over magnesium sulfate and concentrated. Silica gel column chromatography (ethyl acetate/dichloromethane and then ethyl acetate) followed by recrystallization from pentane afforded 0.66 g of a white solid (57%). ^1^H NMR (200 MHz, DMSO*-d*_6_): δ (ppm) = 7.42/7.17 (2d, 4H, Ar-*H*), 3.21 (m, 6H, N-C*H*_2_), 2.15 (s, 3H, Ar-C*H*_3_), 1.78 (m, 3H, C*H*_2_(cycle) + C*H*) , 1.32 (m, 8H, C*H*_2_), 0.93 (t, 6H, C*H*_3_).

**Chain stopper S4.** 2-Ethylhexylamine (8.8 mL, 52 mmol) in dichloromethane (50 mL) was added slowly under a nitrogen atmosphere to a stirred solution of 2,4-toluene diisothiocyanate (5.06 g, 24.5 mmol) in dichloromethane (200 mL, distilled over calcium hydride). After 24 h, the solvent was evaporated. Recrystallization from ethanol/water afforded 7.74 g of a white solid (68%). ^1^H NMR (250 MHz, DMSO-*d*_6_, δ (ppm)): 9.47/9.04 (2s, 2H, Ar-N*H*), 7.52/7.37 (2s, 2H, CH_2_-N*H*), 7.32 (s, 1H, Ar-*H*), 7.21–7.11 (m, 2H, Ar-*H*), 3.40 (m, 4H, N-C*H*_2_), 2.14 (s, 3H, Ar-C*H*_3_), 1.59 (m, 2H, C*H*), 1.25 (m, 16H, C*H*_2_), 0.84 (m, 12H, C*H*_3_). ^13^C NMR (62.5 MHz, DMSO-*d*_6_, δ (ppm)): 181.2/180.4 (*C*=S), 137.0/130.6/130.0/122.6/121.1 (*Ar*), 47.4/47.1 (N-*C*H_2_), 38.4/38.3 (*C*H), 30.5/28.4/23.8/22.6 (*C*H_2_), 17.1 (Ar-*C*H_3_), 14.0/10.7 (*C*H_3_). MS (ESI) = [M-H] 463.4

### Viscometry

Solutions were prepared by stirring at room temperature for at least 1 day prior to use. Capillary viscosity was measured at 25 ± 0.1 °C with an automatic Anton-Paar AMVn viscometer (capillary internal diameter 1.8 mm; ball diameter 1.5 mm). The measurements were performed with an angle of 20° and repeated six times.

#### FTIR spectroscopy

Infrared spectra were recorded on a Nicolet Avatar 320 spectrometer in KBr cells of 0.3 to 2.5 cm path length.

#### SANS

Measurements were made at the LLB (Saclay, France) on the Paxy instrument, at three distance-wavelength combinations to cover the 3 10^−3^ to 0.3 Å^−1^ q-range, where the scattering vector q is defined as usual, assuming elastic scattering, as q = (4π/λ)sin(θ/2), where θ is the angle between incident and scattered beam. The sample diaphragm was 7.6 mm. Collimation was achieved with a diaphragm of 22 mm for a sample – detector distance of 1.5 m, or 16 mm for a sample – detector distance of 3.2 and 6.7 m. Data were corrected for the empty cell signal and the solute and solvent incoherent background. A light water standard was used to normalize the scattered intensities into cm^−1^ units.
